# A multi-phase approach for developing a conceptual model for human resources for health observatory (HRHO) toward integrating data and evidence: a case study of Iran

**DOI:** 10.1186/s12961-023-00994-8

**Published:** 2023-06-01

**Authors:** Zhila Najafpour, Mohammad Arab, Kamran Shayanfard

**Affiliations:** 1 Department of Health Care Management, Public Health Faculty, School of Public Health, Ahvaz Jundishapur University of Medical Sciences, Ahvaz, Iran; 2 School of Public Health, Tehran University of Medical Sciences, Tehran, Iran; 3 Physics and Materials Science Research Unit, University of Luxembourg, Luxembourg, Luxembourg

**Keywords:** Observatory, Human resource for health, Conceptual model, Health workforce

## Abstract

**Background:**

Evidence-informed policymaking on human resources for health (HRH) has been directly linked with health system productivity, accessibility, equity, quality, and efficiency. The lack of reliable HRH data has made the task of planning the HRH more difficult in all settings.

**Aim:**

This study aimed to develop a conceptual model to integrate HRH data and evidence.

**Methods:**

The current study is a mixed-method study conducted in three phases: a rapid literature review, a qualitative phase, and an expert panel. Firstly, the electronic databases were searched up to 2018. Then, in the qualitative phase, semi-structured interviews with 50 experts were conducted. Data analysis was performed using the content analysis approach. After several expert panels, the draft of the model was validated with 15 key informants via two Delphi rounds.

**Results:**

Our proposed model embraces all dominant elements on the demand and supply side of the HRH in Iran. The conceptual model consists of several components, including input (regulatory system, structure, functions), educational system (pre-service and in-service education), health labor market structure, process (technical infrastructure), and output (productions, policymaking process). We considered networking toward sustainable interaction among stakeholders, and also the existence of capacity to integrate HRH information and produce evidence for actions.

**Conclusion:**

The proposed model can be considered a platform for developing a harmonized system based on the HRH data flow to evidence-informed decision-making via networking. We proposed a step-by-step approach for the sustainability of establishing a national human resources for health observatory (HRHO). The proposed HRHO model can be replicable and flexible enough to be used in different context domains.

**Supplementary Information:**

The online version contains supplementary material available at 10.1186/s12961-023-00994-8.

## Introduction

Any health system depends critically on the size and performance of the human resources for health (HRH). It is now evident that in many countries achieving universal health coverage (UHC) requires a considerable increase in the numbers of HRH [1]. Countries still face challenges such as lack of reliable information about the size, composition, mix of skills, and HRH performance [2–4], lack of a national integrated HRH information system [5], noncoherent policies, and inappropriate planning [6, 7]. The mentioned obstacles have made the task of planning and monitoring the HRH more difficult in all settings, while evidence-informed policymaking on HRH has been directly linked with health system productivity, accessibility, equity, quality, and efficiency [8].

Efforts are being made to improve HRH information such as National Health Workforce Accounts (NHWA). The NHWA has been offered a set of appropriate and feasible indicators that help countries to monitor their HRH. While many countries report the indicators, there are uncertainties about the validity of the reported data due to the inherent challenges in data collection at the national level. Tackling the mentioned challenges requires innovative ways to attract stakeholders’ involvement and generate timely and valid information and evidence [9, 10]. The need for comprehensive, standardized, and accurate data on HRH led to the creation of HRH observatories in different countries. The Latin American and Caribbean region was the first to establish an HRH observatory, showing considerable success in advocating for and promoting HRH issues. The countries of this region experienced improvements in aspects such as methods for HR data collection, analysis, assimilation, and utilization. However, the region has also been faced with several challenges, including coping with political instability and maintaining stakeholder commitment as well as using the evidence in policymaking process [11]. In general, the overall success of the countries of the Latin American region led to a tendency to establish national and regional HRH observatories within other regions of the World Health Organization. Consequently, the World Health Organization organized a meeting on health workforce observatories in the Eastern Mediterranean region for improving health workforce information and evidence in the region in 2017 [12].

In the past decades, Iran has faced serious challenges in HRH policymaking and planning. Among other reasons, this can be traced back to the lack of a reliable and integrated HRH information system [13]. To tackle the mentioned issues, setting up an HRH observatory is required.

The observatories can be varied in geographical scope, focus areas, and organizational structures [14]. The observatories should be designed according to each country’s available resources, information communication technology (ICT) infrastructure, and structural capacity [15, 16]. Regarding the different situations concerning HRH management between the countries, the first step to understanding the national context in which HRH programs operate is to create a conceptual model. A conceptual model promotes principles and practices based on an in-depth analysis of the health labor market to understand the driving forces affecting the supply and demand of HRH in countries. In this regard, our research team aimed to develop a comprehensive conceptual framework to design an Iranian National HRH observatory. Our objective was to produce a conceptual model that will help policymakers and researchers to: (i) describe processes and flow of HRH data produced in the health labor market, and (ii) identify key steps toward integrating data and evidence in all related parts and define a common connection to policy dialogue.

## Methods

This is a mixed-method study conducted in three phases: a rapid literature review, a qualitative study, and expert panels.

### Rapid literature review

In the first phase, the rapid literature review was conducted by using online databases, aiming to clarify our first research question: What essential elements should an HRH observatory have? And what are the experiences of other countries? The electronic databases of Medline, Embase, ProQuest, PubMed, Scopus, and Web of Science were searched up to 2018. The research results obtained from the selected databases were entered into the EndNote software; after removing duplicate articles, the titles and the abstracts were screened. After removing irrelevant articles, the full texts of the studies were studied for eligibility. Reference lists of all included studies were screened to identify additional citations for potential eligibility of inclusion. The team of two reviewers (Z.H., K.S.) screened all studies independently. We included comparative studies, systematic reviews, observational studies, and case studies, global, and national reports that have discussed key characteristics of HRH observatories. Also, we assessed the HRH observatories that have already been established without any location limitation (see more details in Additional file 1).

### Qualitative phase

Furthermore, a qualitative assessment of the elements of an HRH observatory was undertaken [15, 16]. This national and subnational assessment builds the foundation of an HRH observatory in Iran and is therefore a key document in the design of the conceptual framework of HRH in our study (see more details in Additional file 2).

#### Model development and validation

Formulating the preliminary conceptual framework is done on the basis of the literature review and the results of the qualitative phase. The HRH observatory conceptual framework has been designed on the basis of the World Health Organization’s workforce lifespan model for human resources for health [17] in three main data layers: data production and collection, data management, and data utilization. In addition, we considered the HRH data flow and their connections and prerequisites during the model development. After two expert panels, the model was prepared and developed by using Microsoft Visio software.

The initial model was assessed via two rounds by using a researcher-made checklist. In the first round, we asked the participants to read the guidance sheet to assess the relevance, implicit connections between different sectors, and the possibility of establishing the HRH observatory (see more details in Additional files 3 and 4).

### Sampling and setting

Model validation was checked with 15 key informants. Participants were selected on the basis of their involvement in health policy, health care management, HRH management, and health information technology fields. All participants invited for the interview had the following inclusion criteria: background in HRH management, health policy, or health information technology, and > 4 years of experience in the related field. Through the review process, 15 interviewees were identified: 6 policymakers in different deputies of the ministry of health and medical education (MOHME), 4 from human resource departments of medical universities, 3 experts of the information system in the MOHME, and 2 professors in the related disciplines. Out of these 15 participants, 5 were physicians, 6 of the directors had a PhD, and 4 had master’s or bachelor’s degree.

### Data collection

**Fig. 1:**
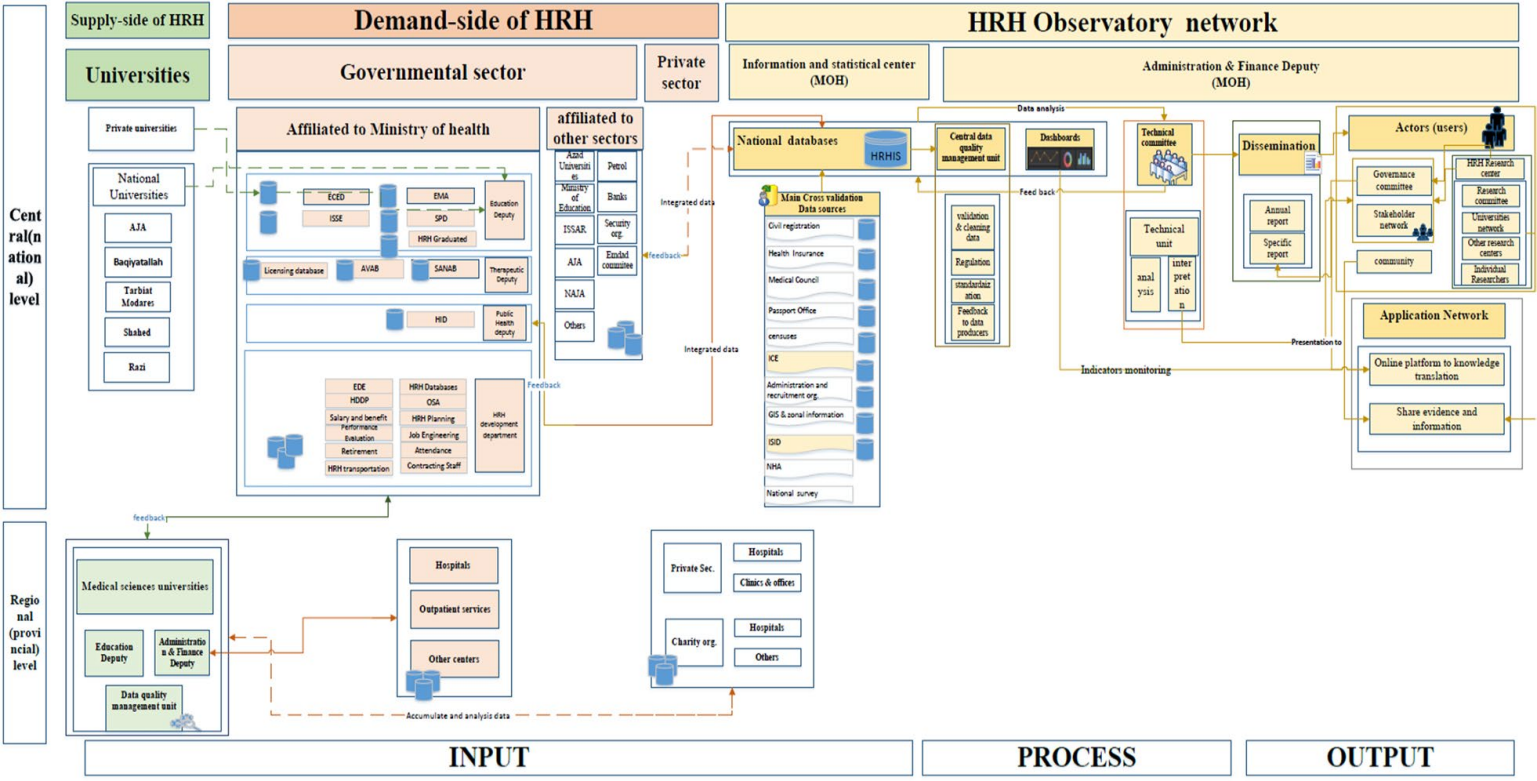
Proposed HRHO conceptual model

The interview guide was prepared on the basis of the model components and literature review [18–21]. The interview guide included contained 15 questions in the 5 domains: 4 items about the data layer, 5 items in the development data layer, 2 items asking about observatory products, 2 items about the model application, and 2 items related to model prerequisites (Table 1). Each item was rated on a five-point Likert-type scale ranging from 1 (strongly agree) to 5 (strongly disagree). Meanwhile, any suggestions about each layer or the proposed model were inquired via an open-ended question. Data gathering was conducted over 2 months, from August to October 2021. At the first round, participants were invited to provide written feedback on the checklist. Next, the first-round results were accumulated for obtaining the rate of agreement for each data layer. The acceptable rate of agreement was considered more than 70% for each layer. The lower score was referred to the second round to acquire the final agreement. For minimizing any potential biases, two researchers from the study team independently assessed the findings derived from the rounds in case of the domains’ associations and contents. Based on the results, the agreement rate in five layers was: 81% in the data layer, 85% in the data development layer, 86% in the data production layer, 88% in the data application layer, and 87% in the data prerequisites. The results present an acceptable percentage of agreement rates in all layers. The finalized version of the conceptual framework was confirmed by the participants in the second round (Fig. 1 and Additional file 3).

**Table 1:** HRHO model validation

Category	Subcategory
Data layer	Data flow between higher academic institutions and health market institutions
	Data flow between education deputy of the MOHME and higher academic institutions
	HRH data sources (inter- and extra-organizations from the MOHME)
	Related health service institutions
Data development	An integrated HRH information system (data warehouse)
	Mechanism to assessing the quality of data and providing feedback
	Communication between information and statistics center of the MOHME and HRHO secretariat
	A specialized center for analyzing, interpreting, translating, and disseminating of information
	HRH indicators for monitoring “ inputs,” “processes,” and “outputs”
Data production	Observatory productions and their connection with users’ networks
	Websites and portals
Data application	Connection between the process of formulation and implementation of HRH policies with the observatory system
	Relations among actors from different parts (national and regional)
Prerequisites	National stakeholders’ network (inter and extra-actors from the MOHME)
	Research and evidence network

## Results

**Fig. 2:**
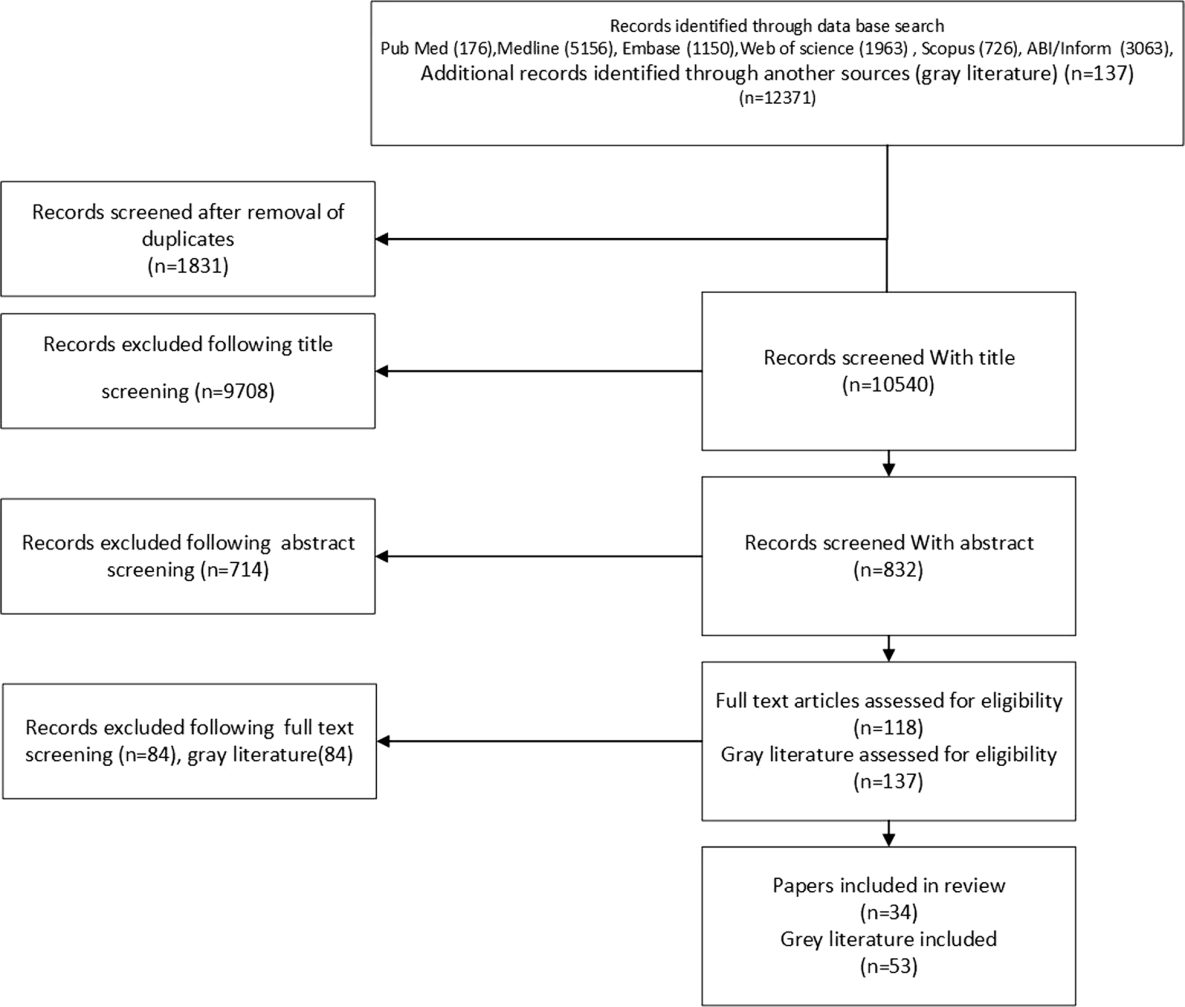
PRISMA diagram

Out of 12,371 articles obtained from the systematic search, a total of 10,540 studies were eligible for title and abstract screening, after removal of duplicates. In total, 118 studies and 137 reports were selected for full-text screening, and finally 34 articles and 53 reports of other sources were included in the analysis of this review (see more details in Additional file 1 and Fig. 2).

Based on our results, the proposed HRHO conceptual framework embraces all dominant elements on demand and supply sides of the HRH system in Iran. The conceptual model consists of several components, including the HRH regulatory system (structure and functions), education system (pre-service and in-service education), and health service delivering, technical infrastructure, and policymaking process.

### General statement of the proposed framework

#### HRHO structure and functions

The proposed HRHO strongly supports the creation of a national HRH observatory under the MOHME stewardship. However, the establishment of HRHO needs to redefine the connections between different deputies of the MOHME and other sectors (i.e., private, army, charity, and social security). The MOHME minister’s political commitment is considered a crucial issue to establish an HRH observatory in Iran.

We design an HRH observatory framework based on cooperation networks including steering and technical committees, stakeholder forum, quality control, and research centers. First, we identified a list of potential stakeholders for the HRH observatory and proposed a structure toward integrity and transparency of decision-making. We suggest a stakeholder forum for involving stakeholders. This forum is created to bring together different stakeholders in the HRH arena for gaining political commitment and support. On the other hand, the steering committee, stakeholder forum, and medical science universities (MSUs) have directly connected as key users through a network. In this network, we attempt to gather different sources of power and knowledge to maximize the opportunities for moving toward evidence-based HRH policymaking, decision-making, planning, actions, and practical research. Meanwhile, an executive secretariat and data controller are suggested as data management centers.

### The supply of HRH

#### Situation analysis

The medical education system in Iran is based on the integration of education in the delivery of services. This means that MSUs, in addition to having a human resource training mission, are also responsible for providing health services to the community. Policymaking in medical education in Iran is granted under an integrated supervision of the MOHME. Education deputy affiliated with the MOHME has the obligation of management and planning of medical education affairs. There are two main types of higher medical education institutions in the country: public and private universities. Most of the HRH, nearly 77.75% of them, are trained directly by the public MSUs affiliated with the MOHME. The share of private universities in training the HRH is 20.5%. Finally, 1.75% of the HRH are trained by centers affiliated with other organizations. In sum, there are currently about 190,000 students studying at 65 state-run universities and colleges of medical sciences and about 60,000 students in 90 private institutions in the field of medical sciences. More than 17,000 faculty members at public centers and about 2000 faculty members at private centers are offering training in the field of health sciences.

#### Problem statement

Students’ information of the MSUs affiliated to the MOHME is available in the deputy of education at the MOHME. At the same time, the so-called deputy has no access to the information of students studying at private universities. Regarding other higher education centers, only information about the AJA University of Medical Sciences, Baqiyatallah University, and Shahed University is available offline to the deputy of education at the MOHME annually. Therefore, it is readily apparent that there is no integrated information system in higher education in Iran. Meanwhile, policymaking in medical education is faced with weaknesses in the decision-making system such as multiplicity of decision-making centers and lack of connection between related deputies in the MOHME.

#### Proposed model

On the basis of the aforementioned situation, we proposed a national system to aggregate student’s data under the deputy of education at the MOHME. Our assessment indicates that all pre-service data on their current enrolled and graduating students are stored in an internal university database called education management database. Although the database contains various relevant information of each educational institution separately, it still needs to be restructured as an integrated training information system (ITIS) at the national level. In the proposed model, ITIS should be connected to the HRH data warehouse to accumulate data on potential HRH available to enter the health market.

### The demand of HRH

#### Situation analysis

Health services in Iran are provided by organizations affiliated with the three main parts: public, private, and charity. The public sector owns the largest share, including 79% hospitals and 26% outpatient services. Organizations affiliated with the public sector are classified into two categories: owned by the MOHME or by other government agencies. In total, 79% of government hospitals belong to the MOHME. The rest (21%) belong to other government organizations [including the Social Security Organization (10%), the Army of the Islamic Republic of Iran (4%), the Martyr and Veterans Affairs Foundation (1%), Islamic Azad University (1%), Islamic Revolutionary Guard Corps (2%), Law Enforcement Force (1%), National Iranian Oil Company (1%) and other organizations (1%)]. Meanwhile, 79% of the country’s health workforce is employed in organizations affiliated with the public sector.

#### Problem statement

On the HRH demand side, there are various health service delivering institutions in the country. Subsequently, large numbers of HRH are working partly in the public sector and partly in another. Although all health-service-delivering institutions record the HRH data in their local HRH databases, there is no integrated national center for accumulating HRH data because of the multiple governance. Thus, integrated information on the HRH in the public and private sectors is not available to provide a comprehensive presentation of the HRH situation in the health market by the MOHME.

#### Proposed model

The proposed HRHO on the HRH demand side consists of two parts because of different stewardship of health-service-delivering institutions: national and province levels.

#### National level

Accordingly, in the proposed model, some measures are featured to integrate HRH information including identifying parallel databases in different parts and establishing a connection among them, as well as creating unique governance in the deputy of management development and resource. Furthermore, regarding other governmental organizations providing health services, it has been suggested that interorganizational agreements be concluded at the ministries level and the chief of relevant organizations agree on exchanging data. In this regard, the use of e-government platforms and the interaction between the information and technology statistics center of the MOHME with the corresponding units in other organizations is of the utmost importance.

#### Province-level

The nongovernmental sector provides a significant part of outpatient health services in the country. The share of organizations affiliated with the private and charity sector in the country includes 21% of hospitals and 74% of outpatient centers. However, due to the lack of unique governance and their scattered distribution throughout the country, the data of full-time HRH employed in the private sector and the public sector HRH working in the private sector (i.e., dual practice) are not integrated. Additionally, to provide a comprehensive picture of HRH in the country, information on all health workforce working in different sectors is necessary. On the same ground, licenses for the establishment, renewal, and accreditation of hospitals and outpatient centers are issued by the MOHME. Therefore, in the proposed model, this capacity was considered to facilitate the transfer of HRH information of the so-called bodies. Accordingly, the MSUs were considered responsible to integrate the data of private and charity sectors within their provinces; and the statistics and information technology departments were proposed to act as the unit supervising the quality of all information registered in these sectors.

### Process of HRH observatory model

Our investigation indicates that the current health resources information system (HRIS) used in the country has a multitude of problems. The main identified barriers were unclear organizational structure and governance, shortage of trained staff, lack of standards among HRH measurement tools and a transfer data center, variety in taxonomy due to different definitions of occupational groups, weak top-down monitoring mechanism, lack of a national plan for HRH data management, and non-use of evidence in policymaking [22]. Several prerequisites needed to be predefined in the proposed model to respond to the barriers. Prerequisites included standardized HRH data elements, integrated different databases in a data warehouse, defined key indicators (KI), and communication mechanisms among all data producers. For this reason, first a national database was designed to accumulate HRHIS with visualization tools to present raw and statistical data for reporting HRH situations and trends, with analysis based on KPI via simple maps and tables. The HRHIS encompasses all HRH data from when professionals enter pre-service education until they leave the health system. Next, all data sources required to connect to the data warehouse were identified and assessed. An online connection between all inter-institutions of the MOHME and an offline connection with all organizations outside the MOHME databases are suggested in the initial phase of launching the HRH observatory. In general, 30 HRH databases were identified, 11 of which were selected to link with the HRH data warehouse. Also, the minimum datasets (MDS) are determined and standardized through the research process [22]. A core dataset should be created to organize collected data from different sources. Next, a quality-control center consists of specialized staff for quality control of the stored data with providing constant feedback to the province centers and also doing routine statistical analysis (i.e., historical and seasonal trends). Meanwhile, a technical committee consists of specialists to perform sophisticated statistical analysis, interpret nonroutine reports based on special requests and policy dialogue, develop standards, and formulate KPI, as well as legal and security issues.

### The output of the HRH observatory

This section is composed of three parts: HRHO products, users, and application networks. We consider several products, including public reporting (HRH country profile) and special reporting (i.e., policy dialogue, research results, analytical report, and raw data). Using information by policymakers depends on timely available information and evidence. Then, comprehensive analysis based on the HRH situation and main challenges that are accompanied to control actions in a user-friendly form can motivate policymakers to use the produced evidence. The critical point is connection between observatory productions and the user’s network. For this issue, we attempted to involve the key HRH decision-makers via networking with building forums in the proposed model. Also, we created a link between the HRHO production and the user’s network on one side and the user’s network and application network on the other side via policy dialogue. Meanwhile, a cooperative research forum is proposed in the users’ network to gather different sections to conduct research based on needs. This forum consists of a specialized HRH research center, MSUs, research centers in the field of HRH in the country, and individual researchers. The cooperative research forum provides research-based evidence to improve informed policymaking to the steering committee and stakeholder forum. In the users’ network a collaboration between different users such as specialist boards, stakeholders, educational institutions and research centers, and researchers is suggested. In the application network, some have planned to share products that include an online observatory platform, knowledge translation database, and a joint meeting between different boards.

## Discussion

Our proposed HRHO model embraces all dominant elements on the demand and supply side of the HRH in Iran. The conceptual model consists of three main phases with a chain of components, that is, the HRH regulatory system, educational system (pre-service and in-service education), and health-service-delivering institutions based on health market structure, technical infrastructure, and HRH policymaking process. We considered networking toward sustainable interaction between key stakeholders, and also the existence capacity for integrating HRH information and producing evidence for actions throughout the model.

Health services are delivered in a wide variety of public and private organizations in Iran. For this reason, the proposed model recommended a hybrid structure in national and province levels on the supply and demand side of the HRH to capture data. HRH planning can be highly compromised and biased because countries do not have access to the nongovernmental sectors. Additionally, considering the need to collect HRH data from multiple sources, we considered some mechanisms to reach a unique governance. For instance, Brazil’s HRH observatory model is a network of academic (universities) institutions, research centers, and the Ministry of Health. It includes about 22 workstations, coordinated by a secretariat made of a staff member of the MOHME and the The Pan American Health Organization’s (PAHO) office in Brasilia [11]. Evidence production for policymakers and designing initiative mechanisms in HRH management have been considered the main functions of the observatory [23, 24]. In Africa and Eastern Mediterranean regions there is a cooperative network comprising national observatories (which bring together the country level stakeholders) and also a regional secretariat with different partners of the region as a steering group. In Sudan, the observatory became the hub for HRH stakeholder coordination and advocacy, developed capacity for data analysis and management, and created an HRH research agenda that should produce a knowledge base for guiding future HRH development [25].

Each observatory requires high-quality, comprehensive, and interoperable HRH data sources to support all policies [26]. Clearly, health workforce monitoring is a prerequisite for strategic planning and policymaking. More attention to workforce data quality, comparability, and availability is required [27]. Unstandardized and disaggregated data on the HRH limit the capacity of countries to systematically and regularly identify gaps in health worker availability [26]. Based on the World Health Organization report, some regions, especially in the Africa region, faced poor statistics at all levels; weak capacity at various levels on methods, technology, standards, and dissemination; insufficient evidence-based knowledge available and used on policy decisions [28]. At the same time, HRH observatory as a political tool could accumulate reliable information about the composition, distribution, size, and location of HRH gaps and help to design effective interventions.

One of the basic infrastructures for HRHO to capture accurate data would be an integrated HRH information system (HRHIS) [29]. The first step of the HRHIS designing process is to undertake a broad-based assessment of the country’s health information systems. Many different types of data are generated, with from the point of view of policymakers and planners some kinds of information being more important than others. In this step, we formulate synthesized indicators based on the policy issues and problems in the country [22]. The next step is describing data requirements and tools at different levels. Matching the data with the most appropriate and cost-effectiveness tool for data generating is an essential function. Then standardization and systematic collection of relevant HRH information are key prerequisites to making the best plan [30]. Correia reported three major problems affecting HRH monitoring in Portugal: insufficient data, the non-use of available data, and the general lack of analysis of the HRH situation [31]. On the basis of our assessment, 30 HRH databases were identified, and 11 databases were specific to the HRH. We assessed all of them and suggested six databases should connect to the HRH warehouse [22]. The Mozambican system is an excellent example of an HRIS built on a local platform with local staff with leadership engagement, intersectoral collaboration, involvement of key stakeholders, and a customized assessment of existing systems and procedures before designing a new system [32]. Finally, data should be synthesized, analyzed, and interpreted within the context of the HRH system’s functioning [33]. To organize collected data from different sources, we proposed quality-control centers at the national and regional levels and a national technical committee in our framework.

Countries face some challenges in the field of data management, including the scarcity and incompleteness of data; variation in definitions of health workforce categories; a lack of data on registered and active workforce; weak data on attrition, international mobility, and unemployment; an absence of information about private sector health workforce, discrepancies between data producers and duplications in reported numbers; underdeveloped digitized records and databases; and a lack of transparency between human resources for health (HRH) stakeholders and political divisions [34]. Several studies made a series of recommendations to overcome the challenges due to establishing an integrated HRH information system. The capacity building of HRH in collection, management, analysis, interpretation, and use of HRH data; the need to connect databases; and strengthening the use of HRH data for policymaking were several proposed recommendations [35, 36]. In the proposed model, HRH data will be collected and accumulated in a national data warehouse, and subsequently, it is planned to control quality of data, analyzing, interpreting, and disseminating the results. In this flow, we attempted to improve interconnections with a restructured link based on networking among the related parts in the field of medical education, health market, research, and policymaking processes. Literature proposed several solutions to improve the stakeholder’s involvement, including a situation analysis of all stakeholders, capacity-building among HRH stakeholders [37], establishing multi-stakeholder committees and working groups for HRH development [14], using the power of the specialist boards [38], formulating national guidelines and standards, and redesigning existing rules and policies [39, 40].

HRH policymaking should be based on problems and functional issues’ systems. We suggest a national forum as a database for HRH research within the Ministry of Health to improve evidence in developing evidence-based HRH policies. In our conceptual framework, by using networks, HRH observatories play a catalytic role in evidence-based policy dialogues and policy development. We proposed a collaboration network between key stakeholders in the field of medical education, health labor market, and health research for policy analysis, research, dialogue, and advocacy. Stakeholder involvement in the governance process brings together principles and values, interests, conflicts, and evidence and information from different levels. The African Health workforce observatory was envisaged to be a cooperative initiative and partnership (public sector, non-profit organization (NGO), academia, professional associations, international and sub-regional organizations, and development partners) to improve human resources development through promoting and facilitating evidence-based policymaking [41]. In Brazil, two significant interventions extracted from the HRH observatory were development of a community of professionals engaged in HRH policy and a marked production of HRH according to national profiles [42]. HRH observatory in Ghana and Malawi has focused on strengthening HRH governance by promoting evidence-based policy and continuous monitoring of indicators as well as disseminating experiences with key stakeholders [43, 44]. The European health observatory framework emphasized “policy capacity” operationalized through formalized structures to connect top-down and bottom-up decision-making, through indicators to measure policy implementation, procedural target setting, training, and policy revision [45]. In 2008, the Council of Australian Governments established Health Workforce Australia (HWA) as the national agency to oversee and progress health workforce reform. One of the main functions of the HWA was to “develop solutions that integrate workforce planning, policy and reform with the complementary reforms to education and training” [46].

We faced several challenges in the course of conceptualizing of an integrated HRH observatory. Some key stakeholders were sensitive to sharing HRH information details. Additionally, we found a considerable fragmentation in different parts of the HRH management system. We attempted to manage the mentioned challenges by applying several actions, including designing a stakeholder forum driven by networking toward sustainable interaction between key stakeholders, and also using all of the existence capacity to integrate HRH information and produce evidence for actions. Also, we considered the MOHME as a strong political position to manage all of the possible challenges in the implementation process. Meanwhile, we assessed policy-level and user-level issues to obtain comprehensive evidence. Finally, we proposed a step‐by‐step approach from the design phase to the rollout phase, to improve sustainability in implementing an HRIO.

## Conclusions

The proposed model is designed on the basis of HRH governance capacity and structure, information and communication technology (ICT) infrastructure, communication mechanism between different sectors, and HRH policymaking process in Iran. Additionally, we considered political conflicts, interactions, existence capacity, and structure of key processes in the field of HRH management. The conceptual model consists of three main phases with a chain of components, including the HRH regulatory system, education system (pre-service and in-service education), and health-service-delivering institutions. We provided technical assistance toward HRH data integration with networking between involved sectors in the HRH planning. We emphasized on HRH development for gaining sustainability to transform information and evidence into actions. Establishing HRH observatory should be conceptualized by contextual needs and based on the mentioned factors in our model to ensure sustainability.

## Supplementary Information

**Additional file 1.** Rapid review phase.

**Additional file 2.** Summary of the qualitative phase’s methods and results.

**Additional file 3.** Model validation phase.

**Additional file 4.** Required information in the model.

## Data Availability

All data analyzed during this study are included in this published article and its Additional files 1, 2, 3, and 4. Any further information is available from the corresponding author on reasonable request.

## Abbreviations

UHC: Universal health coverage
HRIS: HRH information system
MSU: Medical science universities
MOHME: Ministry of Health and Medical Education
HRH: Human resources for health
HIS: Health information system
MDS: Minimum dataset
ICT: Information and communication technology
KI: Key indicator
HRHO: HRH observatory
IO: Input–output
EMA: Education management affairs software
ECED: Evaluation and confirmation of education degree
SPD: Specialist physician’s distribution database
ISSE: Integrated software for specialist student’s education
HID: Health integrated database
OSA: Organizational structure affairs database
EDE: Educational and development employee database
HDDP: HRH Deployment and Distribution Program
ICE: Integrated continued education database for HRH
ISID: Iranian Scientometrics Information Database
NHA: National health accounts
PAHO: Pan American Health Organization
NGO: Non-governmental organization
