# Supplementary material for: A multi-phase approach for developing a conceptual model for human resources for health observatory (HRHO) toward integrating data and evidence: a case study of Iran
Source: Health Res Policy Syst. 2023 Jun 1;21:41. doi: 10.1186/s12961-023-00994-8 (PMC10236653; doi:10.1186/s12961-023-00994-8)
Supplement: Supplementary file 1 — Additional file 1. Rapid review phase. [file 12961_2023_994_MOESM1_ESM.docx]

**Additional file 1: rapid review phase**

**Search strategies**

| Row | Web of science search (11/21/2018) | |
| --- | --- | --- |
| 1 | TI=((Manpower OR “health care manpower” OR “healthcare manpower” OR “medical manpower” OR “health manpower”) OR (staff OR “health care staff” OR “healthcare staff” OR “medical staff” OR “health staff” OR (staff near/2 health)) 0R (Personnel* OR “health care personnel” OR “healthcare personnel” OR “medical personnel” OR “health personnel”) OR (employe* OR “health care employe*” OR “health employe*” OR “healthcare employe*” OR “medical employe*”) OR (worker* OR “healthworker*” OR “health care worker*”or “healthcare worker*” OR “medical worker*”) OR (workforce* OR “health care workforce*” OR “healthcare workforce” OR “health workforce” OR “medical workforce”) OR (”health professional*” OR “health care professional*” or “healthcare professional*” or “medical professional*”) OR “hrm” OR “HR” or (Human near/3 resourc*)) | 135679 |
| 2 | TI=(‘nurs*’ OR ‘doctor*’ OR ‘physician*’ OR ‘midwife*’ OR ‘midwives*’) | 208320 |
| 3 | #1 OR #2 | 341320 |
| 4 | Ts=((track* OR observ* OR metric* OR network OR monitor* OR evaluate* OR assess* OR measure* OR database OR plan* OR framework OR program OR schedul* OR managing OR overse*) NEAR/3 (healthcare OR health* OR medical)) | 325944 |
| 5 | Ts= ((track* OR observ* OR metric* OR network OR monitor* OR evaluate* OR assess* OR measure* OR database OR plan* OR framework OR program OR schedul* OR managing OR overse*) NEAR/3 (personnel* OR employe* OR worker* OR 'workforce*' OR manpower OR staff* OR professional* OR hr)) | 141344 |
| 6 | # 4 OR #5 | 453235 |
| 7 | TS=(“inform* system*” OR “administr* system*” OR “manage* inform* system*”) | 1086736 |
| 8 | #3 AND #6 AND #7 | 1963 |

| Row | SCUPUS (11/27/2018) | |
| --- | --- | --- |
| 1 | SUBJAREA ( medi  OR  nurs  OR  vete  OR  dent  OR  heal  OR  mult )  SUBJAREA ( arts  OR  busi  OR  deci  OR  econ  OR  psyc  OR  soci ) (( manpower  OR  "health care manpower"  OR  "healthcare manpower"  OR  "medical manpower"  OR  "health manpower" )  OR  ( staff  OR  "health care staff"  OR  "healthcare staff"  OR  "medical staff"  OR  "health staff" )  OR  ( personnel*  OR  "health care personnel"  OR  "healthcare personnel"  OR  "medical personnel"  OR  "health personnel" )  OR  ( employe*  OR  "health care employe*"  OR  "health employe*"  OR  "healthcare employe*"  OR  "medical employe*" )  OR  ( worker*  OR  "healthworker*"  OR  "health care worker*"  OR  "healthcare worker*"  OR  "medical worker*" )  OR  ( workforce*  OR  "health care workforce*"  OR  "healthcare workforce"  OR  "health workforce"  OR  "medical workforce" )  OR  ( "health professional*"  OR  "health care professional*"  OR  "healthcare professional*"  OR  "medical professional*" )  OR “hrm” OR “HR” or (Human w/3 resourc*)) | 176074 |
| 2 | TITLE ( nurs*  OR  doctor*  OR  physician*  OR  midwife*  OR  midwives* ) | 25120 |
| 3 | #1 OR #2 | 188875 |
| 4 | SUBJAREA ( medi  OR  nurs  OR  vete  OR  dent  OR  heal  OR  mult )  SUBJAREA ( arts  OR  busi  OR  deci  OR  econ  OR  psyc  OR  soci )  ( ( ( track*  OR  observ*  OR  metric*  OR  network  OR  monitor*  OR  evaluate*  OR  assess*  OR  measure*  OR  database  OR  plan*  OR  framework  OR  program  OR  schedul*  OR  managing  OR  overse* )  W/3  ( healthcare  OR  health*  OR  medical ) )  OR  ( ( track*  OR  observ*  OR  metric*  OR  network  OR  monitor*  OR  evaluate*  OR  assess*  OR  measure*  OR  database  OR  plan*  OR  framework  OR  program  OR  schedul*  OR  managing  OR  overse* )  W/3  ( personnel*  OR  employe*  OR  worker*  OR  'workforce*'  OR  manpower  OR  staff*  OR  professional*  OR  hr ) ) ) | 151490 |
| 5 | SUBJAREA ( medi  OR  nurs  OR  vete  OR  dent  OR  heal  OR  mult )  SUBJAREA ( arts  OR  busi  OR  deci  OR  econ  OR  psyc  OR  soci )  ( ( "inform* system*"  OR  "administr* system*"  OR  "manage* inform* system*" )  W/3  ( healthcare  OR  health*  OR  care  OR  medical ) ) | 2781 |
| 6 | # 3 AND #4 AND #5 | 726 |

| Row | Medline (Ovid) (11/28/2018) | |
| --- | --- | --- |
| 1 | exp health workforce/ | 12234 |
| 2 | exp health Personnel/ | 478837 |
| 3 | exp health employee/ | 31661 |
| 4 | (employe* adj3 (healthcare or Health or care or medical)).tw. | 83098 |
| 5 | (personnel * adj3 (healthcare or Health* or care* or medical*)).tw. | 10437587 |
| 6 | (worker adj3 (healthcare or Health* or care* or medical*)).tw. | 41165 |
| 7 | (workforce* adj3 (healthcare or Health* or care* or medical*)).tw. | 32077 |
| 8 | (manpower adj3 (healthcare or Health* or care* or medical*)).tw. | 4609 |
| 9 | (staff* adj3 (healthcare or Health* or care* or medical*)).tw. | 231490 |
| 10 | (professional* adj3 (healthcare or Health* or care* or medical*)).tw. | 517071 |
| 11 | (hr adj3 (healthcare or Health* or care* or medical*)).tw. | 8474 |
| 12 | (Human adj3 resourc*).tw. | 59073 |
| 13 | ("nurs*" or "doctor*" or "physician*" or "midwife*" or "midwives*").ti. | 619286 |
| 14 | or/1-13 | 10935839 |
| 15 | ((Track* or Observ* or Metric* or Network or Monitor* or Evaluate* or Assess* or Measure* or Database or Plan or Framework or Program or schedul* or managing or project*) adj3 (personnel* or employe* or worker* or workforce* or manpower or staff* or professional* or hr or human resource*)).tw. | 425507 |
| 16 | ((Track* or Observ* or Metric* or Network or Monitor* or Evaluate* or Assess* or Measure* or Database or Plan or Framework or Program or schedul* or managing or project*) adj3 (healthcare or Health* or medical or health care)).tw. | 1073713 |
| 17 | 15 or 16 | 1364087 |
| 18 | ((information system or administration system or management information system) adj3 (healthcare or health* or medical)).tw. | 10310 |
| 19 | 14 and 17 and 18 | 5156 |

| Row | Embase (11/21/2018) | |
| --- | --- | --- |
| 1 | (staff/de OR 'health care personnel'/de OR 'nursing staff'/de OR manpower/de OR 'health care manpower'/de) OR (((personnel* OR employe* OR worker* OR ‘workforce*’ OR manpower OR staff* OR professional* OR hr) NEAR/3 (healthcare OR Health* OR care* OR medical* OR nurs* OR doctor* OR physician* OR midwife* OR midwives*)) OR (Human NEAR/3 resourc*)):ti,ab,kw | 1648091 |
| 2 | (‘nurs*’ OR ‘doctor*’ OR ‘physician*’ OR ‘midwife*’ OR ‘midwives*’): ti | 408430 |
| 3 | # 1 or #2 | 1856894 |
| 4 | ((track* OR observ* OR metric* OR network OR monitor* OR evaluate* OR assess* OR measure* OR database OR plan* OR framework OR program OR schedul* OR managing OR overse*) NEAR/3 (personnel* OR employe* OR worker* OR 'workforce*' OR manpower OR staff* OR professional* OR hr)):ti,ab,kw | 87773 |
| 5 | ((track* OR observ* OR metric* OR network OR monitor* OR evaluate* OR assess* OR measure* OR database OR plan* OR framework OR program OR schedul* OR managing OR overse*) NEAR/3 (healthcare OR health* OR medical)):ti,ab,kw | 304740 |
| 6 | # 4 or #5 | 381514 |
| 7 | ('inform* system*' OR 'administr* system*' OR 'manage* inform* system*') NEAR/3 (healthcare OR health* OR care OR medical) | 36962 |
| 8 | #3 AND #6 AND #7 | 1150 |

| Row | PubMed search (11/21/2018) | |
| --- | --- | --- |
| 1 | health Personnel[MeSH Terms] | 470396 |
| 2 | Health Manpower[MeSH Terms] | 12220 |
| 3 | health professional[MeSH Terms] | 470396 |
| 4 | health workforce[MeSH Terms] | 12220 |
| 5 | human resource[MeSH Terms] | 19066 |
| 6 | (((((health Personnel[MeSH Terms]) OR Health Manpower[MeSH Terms]) OR health professional[MeSH Terms]) OR (((nurs*[Title] OR doctor*[Title] OR physician*[Title] OR midwife*[Title] OR midwives*)[Title]))) OR health workforce[MeSH Terms]) OR human resource[MeSH Terms] | 488918 |
| 7 | ((track*[Text Word] OR observ*[Text Word] OR metric*[Text Word] OR network[Text Word] OR monitor*[Text Word] OR evaluate*[Text Word] OR assess*[Text Word] OR measure*[Text Word] OR database[Text Word] OR plan*[Text Word] OR framework[Text Word] OR program[Text Word] OR schedul*[Text Word] OR managing[Text Word] OR overse*)[Text Word]) | 1413 |
| 8 | ((health information system[Text Word]) OR management information system[Text Word]) OR health administration system[Text Word] | 2232 |
| 9 | # 7 or #8 | 3644 |
| 10 | # 6 and #9 | 176 |

| Row | AB,Inform / proquest (search 12/1/2018) | |
| --- | --- | --- |
| 1 | (TI (employe* near/3 (healthcare* OR Health* OR care* OR medical*)) OR AB(employe* near/3 (healthcare* OR Health* OR care* OR medical*))) OR (TI (workforce* near/3 (healthcare* OR Health* OR care* OR medical*)) OR AB(workforce* near/3 (healthcare* OR Health* OR care* OR medical*))) OR (TI (manpower* near/3 (healthcare* OR Health* OR care* OR medical*)) OR AB(manpower* near/3 (healthcare* OR Health* OR care* OR medical*))) OR (TI (staff near/3 (healthcare* OR Health* OR care* OR medical*)) OR AB(staff near/3 (healthcare* OR Health* OR care* OR medical*))) OR (TI (professional* near/3 (healthcare* OR Health* OR care* OR medical*)) OR AB(professional* near/3 (healthcare* OR Health* OR care* OR medical*))) OR (TI (HR* near/3 (healthcare* OR Health* OR care* OR medical*)) OR AB(HR near/3 (healthcare* OR Health* OR care* OR medical*))) OR (AB (Human resourc*)) OR TI((nurs* OR doctor* OR physician* OR midwife* OR midwives*)) | 1,433,913 |
| 2 | (ti,ab(Track* OR Observ* OR Metric* OR Network OR Monitor* OR Evaluate* OR Assess* OR Measure* OR Database* OR Plan* OR Framework* OR Program* OR schedul* OR manag*)) | 23,411,725 |
| 3 | (((inform[*5]) near/3 (personnel OR employe[*2] OR worker[*2] OR workforce[*2] OR manpower[*2] OR staff OR professional[*2] OR hr* OR human resource[*2] OR healthcare OR Health[*2] OR medical OR nurs[*2] OR doctor[*2] OR physician[*2] OR workforce[*2] OR midwife[*2])) OR ((manage[*5]) near/3 (personnel OR employe* OR worker* OR workforce* OR manpower* OR staff OR professional* OR hr OR human resource[*2] OR healthcare OR Health[*2] OR medical OR nurs[*2] OR doctor[*2] OR physician[*2] OR workforce[*2] OR midwife[*2])) OR ((system[*2]) near/3 3 (personnel OR employe[*2] OR worker[*2] OR workforce[*2] OR manpower[*2] OR staff OR professional[*2] OR hr* OR human resource[*2] OR healthcare OR Health[*2] OR medical OR nurs[*2] OR doctor[*2] OR physician[*2] OR workforce[*2] OR midwife[*2]))) |  |
| 4 | ti,ab (inform* next/3 (personnel OR employe* OR worker* OR workforce* OR manpower* OR staff OR professional* OR hr* OR human resource* OR healthcare OR Health* OR medical or nurs* OR doctor* OR physician* OR workforce* OR midwife*)) OR ti,ab (manage* next/3 (personnel OR employe* OR worker* OR workforce* OR manpower* OR staff OR professional* OR hr* OR human resource* OR healthcare OR Health* OR medical or nurs* OR doctor* OR physician* OR workforce* OR midwife*)) OR ti,ab (system* next/3 (personnel OR employe* OR worker* OR workforce* OR manpower* OR staff OR professional* OR hr* OR human resource* OR healthcare OR Health* OR medical or nurs* OR doctor* OR physician* OR workforce* OR midwife*)) | 2,711 |
| 5 | ((TI (employe* NEAR/3 (healthcare* OR Health* OR care* OR medical*)) OR AB(employe* NEAR/3 (healthcare* OR Health* OR care* OR medical*))) OR (TI (workforce* NEAR/3 (healthcare* OR Health* OR care* OR medical*)) OR AB(workforce* NEAR/3 (healthcare* OR Health* OR care* OR medical*))) OR (TI (manpower* NEAR/3 (healthcare* OR Health* OR care* OR medical*)) OR AB(manpower* NEAR/3 (healthcare* OR Health* OR care* OR medical*))) OR (TI (staff NEAR/3 (healthcare* OR Health* OR care* OR medical*)) OR AB(staff NEAR/3 (healthcare* OR Health* OR care* OR medical*))) OR (TI (professional* NEAR/3 (healthcare* OR Health* OR care* OR medical*)) OR AB(professional* NEAR/3 (healthcare* OR Health* OR care* OR medical*))) OR (TI (HR* NEAR/3 (healthcare* OR Health* OR care* OR medical*)) OR AB(HR NEAR/3 (healthcare* OR Health* OR care* OR medical*)) OR (AB (Human resourc*)) OR TI((nurs* OR doctor* OR physician* OR midwife* OR midwives*))) AND (ti,ab(Track* OR Observ* OR Metric* OR Network OR Monitor* OR Evaluate* OR Assess* OR Measure* OR Database* OR Plan* OR Framework* OR Program* OR schedul* OR manag*))) AND ((ti,ab (inform* next/3 (personnel OR employe* OR worker* OR workforce* OR manpower* OR staff OR professional* OR hr* OR human resource* OR healthcare OR Health* OR medical OR nurs* OR doctor* OR physician* OR workforce* OR midwife*))) OR (ti,ab (manage* next/3 (personnel OR employe* OR worker* OR workforce* OR manpower* OR staff OR professional* OR hr* OR human resource* OR healthcare OR Health* OR medical OR nurs* OR doctor* OR physician* OR workforce* OR midwife*))) OR (system* next/3 (personnel OR employe* OR worker* OR workforce* OR manpower* OR staff OR professional* OR hr* OR human resource* OR healthcare OR Health* OR medical OR nurs* OR doctor* OR physician* OR workforce* OR midwife*))) | 3,554 |

**Included articles and reports**

| Table 1: included articles | | | | | | | |
| --- | --- | --- | --- | --- | --- | --- | --- |
| Row | Author | year | Type of study | **Row** | Author | year | Type of study |
|  | Badr[1] | 2013 | case study |  | Waters[2] | 2013 | qualitative |
|  | Bartza[3] | 2015 | case study |  | Whittaker[4] | 2015 | qualitative |
|  | Alameddine[5] | 2017 | case study |  | Tursunbayeva[6] | 2016 | review |
|  | Buchan[7] | 2011 | case study |  | Riley[8] | 2012 | review |
|  | Chaulagai [9] | 2005 | case study |  | Pierantoni[10] | 2011 | mixed |
|  | Dilu[11] | 2017 | observational |  | Leon[12] | 2015 | review |
|  | Driessen[13] | 2015 | case study |  | Kapoor[14] | 2012 | review |
|  | Pozo-Martin[15] | 2017 | descriptive |  | Hilliard [16] | 2012 | review |
|  | Riley[17] | 2007 | case study |  | Fort[18] | 2017 | qualitative |
|  | Ghosh[19] | 2009 | case study |  | Buchan[20] | 2002 | review |
|  | Gomez[21] | 2018 | case study |  | Ahmadi[22] | 2014 | review |
|  | Ishijima[23] | 2015 | case study |  | Appiagyei[24] | 2014 | qualitative |
|  | Jimenez[25] | 2017 | case study |  | Buchan[26] | 2004 | review |
|  | Kebede[27] | 2014 | case study |  | Bvumbwe[28] | 2018 | review |
|  | Esanga[29] | 2017 | case study |  | Diallo[30] | 2003 | review |
|  | Nigenda[31] | 2011 | case study |  | Spero[32] | 2011 | case study |
|  | Waters[33] | 2017 | quantitative |  | Waters[34] | 2016 | case study |

| Table 2: Included reports | | | |
| --- | --- | --- | --- |
| Row | Publication / Autor by | Year | Title |
|  | WHO[35] | 2016 | Human resources for health country profiles: China |
|  | Wing[36] | 2016 | Health Workforce Analysis Guide, 2016 Edition |
|  | Vries[37] | 2009 | The Impact of Human Resources Information Systems (HRIS) Strengthening |
|  | Dal Poz [38] | 2009 | Handbook-on-monitoring-and-evaluation-of-human-resources |
|  | Necochea [39] | 2013 | Systemic Management of Human Resources for Health |
|  | MOH of Malawi[40] | 2010 | terms of reference of the Malawi health workforce observatory (MHWO) |
|  | Kinsella[41] | 2016 | Health Workforce Planning Models, Tools and Processes in Five Countries: An Evidence Review |
|  | Greenwell[42] | 2018 | Health Information System Strengthening: Standards and Best Practices for Data Sources |
|  | Settle[43] | 2014 | Establishing and Using Data Standards in Health Workforce Information Systems |
|  | Carpio[44] | 2015 | The Health Workforce in Latin America and the Caribbean |
|  | Campos[45] | 2005 | Brazilian-Observatorio-on-Human-Resources-in-Health-2005 |
|  | Broek[46] | 2010 | Policies and practices of countries that are experiencing a crisis in human resources for health: tracking survey |
|  | Bossert[47] | 2007 | Assessing Financing, Education, Management and Policy Context for Strategic Planning of Human Resources for Health |
|  | Badr[48] | 2007 | Establishing an observatory on human resources for health in Sudan |
|  | Alsheikh[49] | 2015 | South Sudan National Policy for Human Resources for Health 2011-2015 |
|  | WHO[50] | 2018 | International Platform on Health Worker Mobility: Evidence, Solutions and Instruments |
|  | WHO[51] | 2017 | MAPPING AND ANALYSIS OF CAPACITY BUILDING INITIATIVES ON HUMAN RESOURCES FOR HEALTH LEADERSHIP |
|  | WHO[52] | 2017 | National Health Workforce Accounts – A Handbook |
|  | WHO[53] | 2017 | health workforce and labour market dynamics in oecd high-income countries: a synthesis of recent analyses and simulations of future supply and requirements |
|  | WHO African Region[54] | 2017 | Side Event 14: National Health workforce Observatories in Africa: Fostering policy dialogue on Health Workforce |
|  | MOH of Sudan[55] | 2016 | National Human Resources for Health Strategic Plan for Sudan |
|  | WHO[56] | 2016 | the health workforce in India: Human Resources for Health Observer Series |
|  | WHO[57] | 2016 | The International Platform on Health Worker Mobility |
|  | WHO[58] | 2016 | Global strategy on human resources for health Workforce 2030 |
|  | Minister of Health OF Portugal [59] | 2015 | National Plan for Health Human Resources Development (NPHHRD) |
|  | WHO[60] | 2015 | Minimum Data Set for Health Workforce Registry: Human Resources for Health Information System |
|  | USAID [61] | 2015 | Strengthening the Health Workforce for Improved Services |
|  | Kenya Ministry Health[62] | 2013 | human resources for health (HRH) assessment report for northern kenya: overview of health workforce distribution across 10 counties |
|  | Global Health Workforce Alliance[63] | 2012 | Rapid Assessment on the effectiveness of the Country Coordination and Facilitation (CCF) process in Sudan, Zimbabwe and Zambia |
|  | WHO[64] | 2011 | Regional consultation on the development of a regional strategy on human resources for health (HRH) 2010–2011 and HRH observatories in the Eastern Mediterranean Region |
|  | WHO[65] | 2012 | assessment tool on the uses and sources for human resources for health (HRH) data WHO country |
|  | WHO[66] | 2008 | Framework and Standards for Country Health Information Systems |
|  | WHO[67] | 2012 | Human Resources for Health Observatories: Contributing to evidence-based policy decisions |
|  | WHO[68] | 2012 | Health workforce governance and leadership capacity in the African Region Review of human resources for health units in the ministries of health |
|  | WHO[69] | 2012 | The labour market for human resources for health in low- and middle-income countries |
|  | Ministry of Health of Ghana[70] | 2011 | Ghana Human Resources for Health Country Profile |
|  | PAHO[71] | 2011 | Handbook for Measurement and Monitoring |
|  | Japan international cooperation agency[72] | 2011 | JICA’s Cooperation on Human Resources for Health |
|  | WHO[73] | 2011 | Evidence-informed Human Resources for Health Policies: the contribution of HRH Observatories |
|  | WHO[74] | 2010 | Human Resources for Health Country Profile- Jordan |
|  | WHO[75] | 2010 | Report of the First Meeting of the Health Workforce Information Reference Group |
|  | WHO[76] | 2010 | Models and tools for health workforce planning and projections |
|  | WHO[77] | 2009 | Human Resources for Health Country Profile of Malawi |
|  | PAHO[78] | 2009 | Core Data Human Resources for Health: Stocks and Flows – Education – Management. Belize |
|  | WHO[79] | 2009 | Toolkit on monitoring health systems strengthening |
|  | WHO[80] | 2008 | Global efforts for strengthening the information and evidence base on human resources for health |
|  | WHO[81] | 2008 | Ghana: implementing a national human resource for health plan |
|  | WHO [82] | 2006 | working to gather for health |
|  | USAID[83] | 2014 | human resources information systems review states India |
|  | WHO[84] | 2008 | Africa Health Workforce Observatory-AHWO |
|  | Pan American Health Organization[85] | 2010 | observatory for human resources in health (strategic model for the re-launch of the initiative) |
|  | WHO[86] | 2010 | National Health Workforce Observatories: In the context of Africa Health Workforce Observatory |
|  | WHO[87] | 2013 | suggested actions for development of national human resources for health observatories in the south- east Asia Region |
