# Supplementary material for: A multi-phase approach for developing a conceptual model for human resources for health observatory (HRHO) toward integrating data and evidence: a case study of Iran
Source: Health Res Policy Syst. 2023 Jun 1;21:41. doi: 10.1186/s12961-023-00994-8 (PMC10236653; doi:10.1186/s12961-023-00994-8)
Supplement: Supplementary file 2 — Additional file 2. Summary of the qualitative phase’s methods and results. [file 12961_2023_994_MOESM2_ESM.docx]

**Additional file 2: summary of the qualitative phase's methods and results**

Participants were selected based on their involvement in HRH management and health information technology fields at national and sub-national levels. we conducted interviews with 30 in-filed experts: four policy makers in educational, treatment and health deputies of the ministry of health and medical education (MOHME), six from human resource departments affiliated to the deputy for management development, resources and planning in the MOHME who are responsible for human resource management, five from human resource departments of medical universities, three experts of the information system in the MOHME, five professors HRH management or health government and health technology, four staff experts of the human resource and information technology and statistics departments in the MOHME and medical universities.  The interviews were semi-structured with 23 open-ended questions. The interview guide included the following dimension: organizational structure, HRH data sources, key stakeholders, HRH indicators, data management. All interviews were recorded and then were transcribed verbatim. Transcripts were provided to the participants for validation. In the current study, the content analysis method was used to identify categories and subcategories in participants' descriptions. Coding was done using an inductive approach. Our coding process was developed in multiple stages. First, a team consisting of two researchers read the transcripts line by line and selected the codes separately (ZN, KS). After that, the codes were divided into categories and subcategories. Third, they met to check and discuss the codes. Next, all researchers reviewed the coded text to identify the concepts and relationships between themes, categories, and sub-categories.

| Table 3: Qualitative phase's results | | |
| --- | --- | --- |
| Dimension | **Theme** | **Category** |
| HRH observatory framework | HRH observatory structure | National structure Vs Regional structure |
|  | HRH observatory secretariat | Central organization level Vs regional organization level |
| Partnership | Stakeholders | Key stakeholders’ network |
|  | communication mechanism | Central organizational interaction |
|  |  | indirect organizational interaction |
|  | HRH educational system | Communication mechanism of the HRH Educational System with HRH observatory |
| Technical perquisite in implementing HRH observatory | Develop a strategy roadmap | Priorities in the field of HRH |
|  | Key indicators | Focus on HRH lifespan |
|  |  | HRH challenges |
|  | HRH Information systems | Data elements requirements |
|  |  | Hardware and software requirements |
| Data management | Data gathering and quality control | Identification of potential data sources |
|  |  | Interaction between data sources (inter- and extra-organizations from the MOH) |
|  |  | Data accumulation and integration |
|  |  | Data Quality control in HRH information databases |
|  | Analysis and dissemination | Analysis |
|  |  | observatory products |
|  |  | Dissemination |
| Evidence informed policy making | HRH evidence production | National HRH research center |
|  |  | Network of researchers and decision-makers |
|  | Utilization network | Policy makers |
|  |  | Researchers |
|  |  | Public |
