# Supplementary material for: A multi-phase approach for developing a conceptual model for human resources for health observatory (HRHO) toward integrating data and evidence: a case study of Iran
Source: Health Res Policy Syst. 2023 Jun 1;21:41. doi: 10.1186/s12961-023-00994-8 (PMC10236653; doi:10.1186/s12961-023-00994-8)
Supplement: Supplementary file 3 — Additional file 3. Model validation phase. [file 12961_2023_994_MOESM3_ESM.docx]

**Additional file 3: Model validation phase**

**Interview guild**

1.      Is there a data flow between higher academic institutions (supply-side) and health market institutions (demand-side) in the model?

2.      Is there a connection between the educational policy-making center (education deputy of the MOHME) and academic institutions in the model??

3.      Is there all of the related HRH data sources (inter and extra- of MOHME) in the model? What is your opinion about their connection with the data warehouse?

4.      Is there all of the related health service institutions (i.e., public, private, army, charity, and others) in the health market in the model?

5.      Is there a data warehouse to integrate the HRH data in different sectors?

6.      Is a mechanism to assess the quality of data and provide feedback defined?

7.      Is a suitable position for the information and statistics center of the MOHME and HRHO secretariat defined?

8.      Is there a specialized center for analyzing, interpreting, translating and disseminating information in the model?

9.      Are the HRH indicators for monitoring " inputs", "processes" and "outputs" defined?

10.   Is the connection between HRH observatory productions with the users’ networks correctly defined?

11.   Is a connection between the process of formulation and implementation of HRH policies with the observatory system properly defined?

12.   Is it correctly defined the relations among actors from different parts (national and regional) of the country?

13.   Is there a network of internal and external stakeholders in the model?

14.   Has it developed a mechanism for HRH evidence production in the model?

15.   Is there a connection between research network and evidence-based practice in the model?

16.   Do you have any suggestions or additional comments about the proposed model?

**Model validation method**

After formulating the initial model and its protocol, each participant evaluated the model based on the mentioned items in the table1. Then they scored each item (on a Likert scale), and finally, their opinions were gathered around each item.  Each layer had a different number of questions.  To obtain the agreement rate, we equalized the score of all areas to compare better. The score of all areas was converted into a number between 0 and 100. For this purpose, the questions were converted to a number between 0 and 100 using the following formula, and then the average of these questions was calculated for the relevant area. The final score of each question = 100 * (current score - minimum possible score) divided by the possible score range.

Based on the results, the agreement rate in five layers was: 81 percent in the data layer, 85 percent in the data development layer, 86 percent in the data production layer, 88 percent in data application layer, and 87 percent in data prerequisites. Results present an acceptable percentage of agreement rates in all layers. The finalized version of the conceptual framework confirmed by the participants in the second round.
