# Supplementary material for: A multi-phase approach for developing a conceptual model for human resources for health observatory (HRHO) toward integrating data and evidence: a case study of Iran
Source: Health Res Policy Syst. 2023 Jun 1;21:41. doi: 10.1186/s12961-023-00994-8 (PMC10236653; doi:10.1186/s12961-023-00994-8)
Supplement: Supplementary file 4 — Additional file 4. Required information in the model. [file 12961_2023_994_MOESM4_ESM.docx]

**Additional file 4: required information in the Model**

**List of using abbreviations in the model**

- HRIS: HRH Information System
- HRH: Human Resources for Health
- HIS: Health Information System
- HRHO: HRH Observatory
- EMA: Education management affairs software
- ECED: Evaluation and Confirmation of education degree
- SPD: Specialist physician’s distribution database
- ISSE: Integrated software for specialist student’s education
- HID: Health integrated database
- OSA: Organizational Structure Affairs Database
- EDE: Educational and Development Employee Database
- HDDP: HRH Deployment and Distribution Program
- ICE: Integrated continues education database for HRH
- AVAB: HRH information in the treatment deputies
- SANAB: HRH information in hospitals
- ISID: Iranian Scientometrics Information Database
- NHA: National health accounts

**Model guidance**

| Communication and feedback |  |
| --- | --- |
| inter-institutions of the MOH |  |
| organizations outside the MOH |  |
| Database |  |
| Grouping units based on types of ownership |  |
| Related units based on real |  |
| Output |  |

**References:**

1. Badr, E., N. Mohamed, M.M. Afzal, and K.M. Bile, *Strengthening human resources for health through information, coordination and accountability mechanisms: the case of the Sudan.* Bull World Health Organ, 2013. **91**(11): p. 868-73.

2. Waters, K.P., A. Zuber, R.M. Willy, R.N. Kiriinya, A.N. Waudo, T. Oluoch, . . . P.L. Riley, *Kenya's health workforce information system: a model of impact on strategic human resources policy, planning and management.* International journal of medical informatics, 2013. **82**(9): p. 895-902.

3. Bartz, C.C., N.R. Hardiker, and A. Coenen, *Toward a Global eHealth Observatory for Nursing.* Studies in Health Technology and Informatics, 2015. **216**: p. 1114-1114.

4. Whittaker, M., N. Hodge, R.E. Mares, and A. Rodney, *Preparing for the data revolution: identifying minimum health information competencies among the health workforce.* Human resources for health, 2015. **13**: p. 1-12.

5. Alameddine, M., N. Chamoun, R. Btaiche, N. El Arnaout, N. Richa, and H. Samaha-Nuwayhid, *The workforce trends of nurses in Lebanon (2009–2014): A registration database analysis.* PLoS One, 2017. **12**(8): p. e0182312.

6. Tursunbayeva, A., C. Pagliari, R. Bunduchi, and M. Franco, *Human resource information systems in health care: protocol for a systematic review.* JMIR research protocols, 2015. **4**(4): p. e4922.

7. Buchan, J., I. Fronteira, and G. Dussault, *Continuity and change in human resources policies for health: lessons from Brazil.* Human resources for health, 2011. **9**(1): p. 1-13.

8. Riley, P.L., A. Zuber, S.M. Vindigni, N. Gupta, A.R. Verani, N.L. Sunderland, . . . H. Patrick, *Information systems on human resources for health: a global review.* Human resources for health, 2012. **10**(1): p. 1-12.

9. Chaulagai, C.N., C.M. Moyo, J. Koot, H.B. Moyo, T.C. Sambakunsi, F.M. Khunga, and P.D. Naphini, *Design and implementation of a health management information system in Malawi: issues, innovations and results.* Health policy and planning, 2005. **20**(6): p. 375-384.

10. Pierantoni, C.R. and A.C.P. Garcia, *Human resources for health and decentralization policy in the Brazilian health system.* Human Resources for Health, 2011. **9**: p. 1-6.

11. Dilu, E., M. Gebreslassie, and M. Kebede, *Human Resource Information System implementation readiness in the Ethiopian health sector: a cross-sectional study.* Human resources for health, 2017. **15**: p. 1-10.

12. Leon, N., L. Brady, A. Kwamie, and K. Daniels, *Routine Health Information System (RHIS) interventions to improve health systems management.* Cochrane Database Syst Rev, 2015. **12**(1).

13. Driessen, J., D. Settle, D. Potenziani, K. Tulenko, T. Kabocho, and I. Wadembere, *Understanding and valuing the broader health system benefits of Uganda’s national Human Resources for Health Information System investment.* Human Resources for Health, 2015. **13**(1): p. 1-9.

14. Kapoor, B. and J. Sherif, *Global human resources (HR) information systems.* Kybernetes, 2012. **41**(1/2): p. 229-238.

15. Pozo-Martin, F., A. Nove, S.C. Lopes, J. Campbell, J. Buchan, G. Dussault, . . . A. Siyam, *Health workforce metrics pre-and post-2015: a stimulus to public policy and planning.* Human resources for health, 2017. **15**(1): p. 1-16.

16. Hilliard, T.M. and M.L. Boulton, *Public health workforce research in review: a 25-year retrospective.* American journal of preventive medicine, 2012. **42**(5): p. S17-S28.

17. Riley, P.L., S.M. Vindigni, J. Arudo, A.N. Waudo, A. Kamenju, J. Ngoya, . . . M. Kelley, *Developing a nursing database system in Kenya.* Health Services Research, 2007. **42**(3p2): p. 1389-1405.

18. Fort, A., C. Ng, and E. Nicholson, *Guidelines for developing monitoring and evaluation plans for human resources for health.* IntraHealth International. July, 2015.

19. Ghosh, B., *Health workforce development planning in the Sultanate of Oman: a case study.* Human Resources for Health, 2009. **7**(1): p. 1-15.

20. Buchan, J. and M.R. Dal Poz, *Skill mix in the health care workforce: reviewing the evidence.* Bulletin of the World health Organization, 2002. **80**(7): p. 575-580.

21. Gomez, C.M., L.C.F.d. Vasconcellos, and J.M.H. Machado, *A brief history of worker’s health in Brazil’s Unified Health System: progress and challenges.* Ciência & Saúde Coletiva, 2018. **23**: p. 1963-1970.

22. Ahmadi, M., S. Damanabi, and F. Sadoughi, *A Comparative Study of the proposed models for the components of the national health information system.* Acta Informatica Medica, 2014. **22**(2): p. 115.

23. Ishijima, H., M. Mapunda, M. Mndeme, F. Sukums, and V.S. Mlay, *Challenges and opportunities for effective adoption of HRH information systems in developing countries: national rollout of HRHIS and TIIS in Tanzania.* Human resources for health, 2015. **13**(1): p. 1-14.

24. Appiagyei, A.A., R.N. Kiriinya, J.M. Gross, D.N. Wambua, E.O. Oywer, A.K. Kamenju, . . . M.F. Rogers, *Informing the scale-up of Kenya’s nursing workforce: a mixed methods study of factors affecting pre-service training capacity and production.* Human Resources for Health, 2014. **12**(1): p. 1-10.

25. Jimenez, M.M., A.L. Bui, E. Mantilla, and J.J. Miranda, *Human resources for health in Peru: recent trends (2007–2013) in the labour market for physicians, nurses and midwives.* Human Resources for Health, 2017. **15**: p. 1-7.

26. Buchan, J. and J. Sochalski, *The migration of nurses: trends and policies.* Bulletin of the World Health Organization, 2004. **82**(8): p. 587-594.

27. Kebede, D., C. Zielinski, P.E. Mbondji, M. Piexoto, W. Kouvividila, and L.G. Sambo, *The African Health Observatory and national health observatories as platforms for strengthening health information systems in sub-Saharan Africa*. 2014, SAGE Publications Sage UK: London, England. p. 6-9.

28. Bvumbwe, T. and N. Mtshali, *Nursing education challenges and solutions in Sub Saharan Africa: an integrative review.* BMC nursing, 2018. **17**(1): p. 1-11.

29. Likofata Esanga, J.-R., C. Viadro, L. McManus, J. Wesson, N. Matoko, E. Ngumbu, . . . D. Trudeau, *How the introduction of a human resources information system helped the Democratic Republic of Congo to mobilise domestic resources for an improved health workforce.* Health policy and planning, 2017. **32**(suppl_3): p. iii25-iii31.

30. Diallo, K., P. Zurn, N. Gupta, and M. Dal Poz, *Monitoring and evaluation of human resources for health: an international perspective.* Human resources for health, 2003. **1**: p. 1-13.

31. Nigenda, G.G., M.H. Machado, F.F. Ruiz, V.V. Carrasco, P.P. Moliné, and S.S. Girardi, *Towards the construction of health workforce metrics for Latin America and the Caribbean.* Human resources for health, 2011. **9**(1): p. 1-9.

32. Spero, J.C., P.A. McQuide, and R. Matte, *Tracking and monitoring the health workforce: a new human resources information system (HRIS) in Uganda.* Human resources for health, 2011. **9**: p. 1-10.

33. Waters, K.P., A. Zuber, T. Simbini, Z. Bangani, and R.S. Krishnamurthy, *Zimbabwe’s Human Resources for Health Information System (ZHRIS)—an assessment in the context of establishing a global standard.* International Journal of Medical Informatics, 2017. **100**: p. 121-128.

34. Waters, K.P., M.E. Mazivila, M. Dgedge, E. Necochea, D. Manharlal, A. Zuber, . . . A.E. Vergara, *eSIP-Saúde: Mozambique’s novel approach for a sustainable human resources for health information system.* Human Resources for Health, 2016. **14**(1): p. 1-10.

35. *WHO Library Cataloguing-in-Publication Data: Human resources for health country profiles: China.* WHO, 2016.

36. Wing, P., D. Armstrong, G. Forte, and J. Moore, *Health workforce analysis guide, health workforce technical assistance center.* Center for Health Workforce Studies, School of Public Health. SUNY Albany. <http://www>. healthworkforceta. org/wpcontent/uploads/2016/10/Health-Workforce-Analysis-Guide_2016-Edition. pdf, 2016.

37. de Vries, D., D. Settle, and P. McQuide, *The impact of human resources information systems (HRIS) strengthening.* The Capacity Project Legacy Series, 2009. **6**.

38. Dal Poz, M.R., N. Gupta, E. Quain, A.L. Soucat, and W.H. Organization, *Handbook on monitoring and evaluation of human resources for health: with special applications for low-and middle-income countries*. 2009: World Health Organization.

39. Necochea, E., M. Badlani, and D. Bossemeyer, *Systemic management of human resources for health: an introduction for health managers*. 2013: Jhpiego.

40. *terms of reference of the Malawi health workforce observatory (MHWO).* Malawi MOH, 2010.

41. Kinsella, S. and R. Kiersey, *Health workforce planning models, tools and processes in five countries: an evidence review.* Dublin: Health Research Board, 2016.

42. Greenwell, F. and S. Salentine, *Health information system strengthening: Standards and best practices for data sources.* Chapel Hill: MEASURE Evaluation, University of North Carolina, 2018: p. 58-63.

43. Settle, D., M.W. Lwetabe, A. Puckett, and C. Leitner, *Establishing and Using Data Standards in Health Workforce Information Systems.* 2014.

44. Carpio, C. and N.S. Bench, *The health workforce in Latin America and the Caribbean: an analysis of Colombia, Costa Rica, Jamaica, Panama, Peru, and Uruguay*. 2015: World Bank Publications.

45. Campos, F.E. and V. Hauck, *Networking Collaboratively: The Brazilian Observatorio on Human Resources in Health*. 2005: European Centre for Development Policy Management.

46. Broek, A.v.d., F.G.l. Gedik, M.R. Dal Poz, M. Dieleman, and W.H. Organization, *Policies and practices of countries that are experiencing a crisis in human resources for health: tracking survey*. 2010: World Health Organization.

47. Bossert, T.J., T. Bossert, T. Bärnighausen, D. Bowser, A. Mitchell, and G. Gedik, *Assessing financing, education, management and policy context for strategic planning of human resources for health*. 2007: World Health Organization.

48. Badr, E.E., *Establishing an observatory on human resources for health in Sudan.* Report prepared for the World Health Organization and Federal Ministry of Health of the Republic of Sudan, 2007.

49. Alsheikh, G.M., *SOUTH SUDAN NATIONAL STRATEGIC PLAN FOR HUMAN RESOURCES FOR HEALTH 2011-2015.*

50. *International Platform on Health Worker Mobility: Evidence, Solutions and Instruments.* WHO, 2018

51. Organization, W.H., *Mapping and analysis of capacity building initiatives on human resources for health leadership.* 2017.

52. Organization, W.H., *National health workforce accounts: a handbook.* 2017.

53. Organization, W.H., *Health workforce and labor market dynamics in OECD high-income countries: a synthesis of recent analyses and simulations of future supply and requirements.* 2017.

54. Dodder B, M., *National Health workforce Observatories in Africa: Fostering policy dialogue on Health Workforce.* WHO African Region, 2017.

55. Organization, W.H., *National human resources for health strategic plan for Sudan, 2012-2016 Geneva: WHO;[cited 2017 September 29]*.

56. Anand, S., V. Fan, and W.H. Organization, *The health workforce in India*. 2016: World Health Organization.

57. *The International Platform on Health Worker Mobility: Elevating dialogue, knowledge and international cooperation.* WHO, 2016.

58. Organization, W.H., *Global strategy on human resources for health: workforce 2030.* 2016.

59. *National Plan for Health Human Resources Development (NPHHRD): 2008-2015.* Institute of Hygiene and Tropical Medicine, Lisbon, Portugal, 2016.

60. Organization, W.H., *Minimum Data Set for Health Workforce Registry: Human Resources for Health Information System*. 2015, Geneva, Switzerland: WHO Press.

61. *Strengthening the Health Workforce for Improved Services: Results and Lessons Learned from CapacityPlus 2009-2015.* USAID, 2016.

62. Miseda, M.H., S.O. Were, C.A. Murianki, M.P. Mutuku, and S.N. Mutwiwa, *The implication of the shortage of health workforce specialist on universal health coverage in Kenya.* Human resources for health, 2017. **15**: p. 1-7.

63. *Country CoordInatIon and FaCIlItatIon (CCF) PRINCIPLES AND PROCESS.* Global Health Workforce Alliance, 2006.

64. Organization, W.H., *Report on the regional consultation on the development of a regional strategy on human resources for health [HRH] 2010–2011 and HRH observatories in the Eastern Mediterranean Region, Tunis, Tunisia, 21–24 September 2010*. 2011.

65. Organization, W.H., *WHO Country Assessment Tool on the uses and sources for human resources for health (HRH) data.* 2012.

66. Organization, W.H., *Framework and standards for country health information systems*. 2008: World Health Organization.

67. Gedik, G. and M. Dal Poz, *Human Resources for Health Observatories: contributing to evidence-based policy decisions.* Hum Res Health Obs, 2012. **10**.

68. Nyoni, J. and F.G. Gedik, *Health workforce governance and leadership capacity in the African region: review of human resources for health units in the ministries of health.* 2012.

69. Scheffler, R., T. Bruckner, and J. Spetz, *The labour market for human resources for health in low-and middle-income countries.* Human resources for health observer, 2012. **11**.

70. Health., M.o., *Ghana human resources for health country profile*. 2011, Ghana Health workforce Observatory Kumasi, Ghana.

71. *andbook for Measurement and Monitoring Indicators of the Regional Goals for Human Resources for Health: A Shared Commitment.* Pan American Health Organization, 2011.

72. *JICA’s Cooperation on Human Resources for Health.* Japan International Cooperation Agency (JICA), 2011.

73. *evidence informed human resources for health policies : the contribution of hrh observatories.* WHO, 2011.

74. Ajlouni, M., *Human resources for health country profile-Jordan.* World Health Organization, 2010.

75. Organization, W.H., *Report of the first meeting of the Health Workforce Information Reference Group. 2010*. 2014.

76. Organization, W.H., *Models and tools for health workforce planning and projections.* 2010.

77. Observatory, A., *Human Resources for Health Country Profile Malawi*. 2009, Republic of Congo Brazzaville.

78. *Core Data Human Resources for Health: Stocks and Flows – Education – Management. Belize2009: Tracking Regional Goals for human resource for health A Shared Commitment.* Pan American Health Organization, 2009.

79. Organization, W.H., *Toolkit on monitoring health systems strengthening.* WHO. 2008b. WHO, 2009: p. 17-3.

80. *Global efforts for strengthening the information and evidence base on human resources for health.* WHO, 2008.

81. *Ghana: Implementing a national human resources for health plan: GHWA Task Force on Scaling Up Education and Training for Health Workers.* WHO, 2008.

82. Organization, W.H., *Working together for health: the World health report 2006: policy briefs*. 2006: World Health Organization.

83. Shukla, M., S. Verma, M. Narayanan, and D. Potenziani, *Human Resources Information Systems (HRIS): A Review across States of India.* IntraHealth International, Inc, 2014.

84. Organization, W.H., *Africa Health Workforce Observatory-AHWO.* 2008.

85. *OBSERVATORY FOR HUMAN RESOURCES IN HEALTH (Strategic Model for the Re-launch of the Initiative)*

Pan American Health Organization, 2010.

86. World health organization, *National Health Workforce Observatories In the context of Africa Health Workforce Observatory.* 2010.

87. Organization, W.H., *Suggested actions for development of national human resources for health observatories in the South-East Asia Region*. 2013, WHO Regional Office for South-East Asia.
